# Unlocking Thermal Insulation Performance and Mechanisms in Decoration Waste Aerogel Mortar: A Multi-Factor Study on Paste-to-Aggregate Ratio, Silica Aerogel Content, and Air-Entraining Agent Dosage

**DOI:** 10.3390/ma19163550

**Published:** 2026-08-21

**Authors:** Tianyu Ma, Hui Liu, Yushi Gu, Xiang Guo, Minqi Hua, Zhongmeng Gao, Jun Cui, Zhihao Zhou

**Affiliations:** 1 School of Urban Construction, Changzhou University, Changzhou 213164, China; 2 School of Civil Engineering & Architecture, Wuhan University of Technology, Wuhan 430070, China; 3 School of Architecture and Engineering, Huanggang Normal University, Huanggang 438000, China

**Keywords:** silica aerogel mortar, decoration waste, paste-to-aggregate ratio, air-entraining agent, thermal insulation performance

## Abstract

To improve the resource utilization of decoration waste and enhance the thermal insulation performance of building mortar, this study designed and prepared decoration waste aerogel mortar (DWAM) using decoration waste recycled fine aggregate (DWRA) combined with silica aerogel. The effects of paste-to-aggregate ratio (30/70, 35/65, and 40/60), aerogel content (60–100 vol.%), and air-entraining agent (AEA) dosage (0.1–0.5 wt%) on workability, dry density, mechanical strength, and thermal conductivity were systematically investigated. Microstructural evolution and pore characteristics were analyzed using scanning electron microscopy (SEM) and X-ray computed tomography (X-CT). Results showed that increasing the paste-to-aggregate ratio improved workability and mechanical strength, while aerogel and AEA incorporation significantly reduced thermal conductivity at the expense of strength. An optimum mix with a paste-to-aggregate ratio of 40/60, aerogel content of 80 vol.%, and AEA dosage of 0.4 wt% achieved a dry density of 623.8 kg/m^3^, compressive strength of 1.27 MPa, flexural strength of 0.61 MPa, and thermal conductivity of 0.0784 W/(m·K). X-CT revealed that closed micropores enhanced thermal insulation by disrupting heat transfer. The developed DWAM offers balanced workability, mechanical properties, and thermal insulation, demonstrating strong potential as a sustainable material for building applications.

## 1. Introduction

Continuous improvement in living standards has driven growing demand for residential decoration, leading to a sharp increase in decoration waste (DW). The sources of DW are diverse, and the waste volume fluctuates markedly due to factors such as architectural style, decoration materials, scale, and challenges in recycling and transportation, making disposal difficult. Currently, the comprehensive recycling rate of DW in China is less than 40%. Most waste is simply landfilled, and only a small fraction of materials (such as bricks and wood) is reused [1,2]. This practice not only causes severe resource waste but also imposes a heavy environmental burden. Meanwhile, the annual energy consumption of the building sector accounts for more than 40% of the national total energy consumption, hindering green and sustainable development. Efficient energy conservation has become an urgent issue to be addressed in the building sector [3,4].

Given the sharp increase in construction and demolition waste, researchers have crushed such waste into recycled aggregate (RA) to produce recycled aggregate concrete (RAC) and recycled mortar (RM) to reduce energy consumption and promote sustainability [5,6]. Studies show that replacing natural aggregate (NA) with RA significantly reduces the thermal conductivity of cement-based materials. Bravo et al. [7] reported a 42% reduction when 100% recycled fine aggregate (RFA) replaced NA. Sargam et al. [8] observed a 40% decrease under the same replacement ratio. Kim et al. [9] and Zhu et al. [10] found that thermal conductivity decreased with increasing RA replacement, reaching 70–85% of natural concrete at 100% replacement, mainly due to the higher porosity of RA [8,11]. DW, a typical component of construction waste, remains a challenging issue for resource utilization. Decoration waste recycled fine aggregate (DWRA) is lightweight with high porosity and water absorption [12,13] that reduce heat transfer and thermal convection in mortar [14], giving DWRA potential for preparing insulating mortar. However, the reduction in thermal conductivity from DWRA alone is limited and cannot meet insulating mortar standards. Thus, introducing other highly porous materials is needed for synergistic optimization to obtain RM with desired thermal insulation.

Currently, thermal insulation materials in construction mainly include expanded perlite, rock wool, and ceramics [15]. Researchers are actively exploring more feasible alternatives to reduce building energy consumption [16,17]. In recent years, aerogel has attracted attention due to its ultra-high porosity, extremely low density, and ultra-low thermal conductivity, with applications in aerospace, military, and construction. Incorporating silica aerogel particles into cement-based materials offers new possibilities for lightweight insulating composites [18]. Liu et al. [19] prepared silica aerogel mortar by replacing fine aggregate with aerogel using an equal volume method. With 60% KH550-modified aerogel, the thermal conductivity reached as low as 0.0859 W/(m·K). Zhu et al. [20] developed an aerogel insulating mortar to enhance high-temperature resistance of high-performance concrete under tunnel fire conditions. A 40–50 mm thick aerogel mortar layer effectively prevented fire-induced spalling. Moreover, mortar with 80% aerogel exhibited an initial thermal conductivity of 0.062 W/(m·K), tensile bond strength of 0.965 MPa, and compressive shear strength of 943.56 kPa and remained compliant with Type II insulating mortar standards after 80 hygrothermal cycles and fire simulations [21].

In addition to introducing low-thermal-conductivity aerogel, the paste-to-aggregate ratio (P/A) and admixtures also affect the thermal insulation performance of mortar. A lower P/A increases the volume of aerogel replacing aggregate, leading to higher porosity and reduced density and thermal conductivity, thereby improving insulation. Admixtures not only enhance workability, strength, and durability but also influence insulation. Air-entraining agents (AEAs) generate numerous closed micropores within cement-based materials, reducing solid heat transfer efficiency and thus improving thermal insulation. Kim et al. [22] found that AEAs increased the workability of fresh concrete, reduced pore size in hardened concrete, and optimized pore distribution, with thermal conductivity decreasing linearly as porosity increased. Lu et al. [23] reported that the density and thermal conductivity of aerogel-cement composites are highly correlated with porosity: when porosity increased from 42.8% to 76.6%, thermal conductivity dropped from 0.353 W/(m·K) to 0.066 W/(m·K). Abbas et al. [24] observed that cement composite incorporating 50% silica aerogel exhibited a porosity of 29.83%, and porosity is closely related to density, compressive strength, and thermal conductivity [25]. Previous studies have quantitatively demonstrated the thermal insulation potential of waste-derived and lightweight constituents in cementitious mortars [26]. For example, waste glass powder and foamed waste–plastic aggregates have been reported to reduce both mortar density and thermal conductivity [27], while the combined incorporation of recycled polyethylene terephthalate (PET) and silica aerogel can achieve a more pronounced reduction in thermal conductivity [28]. Representative results from previous studies are summarized in Table 1. Nevertheless, limited attention has been given to the combined effects of construction waste-derived materials, silica aerogel, and controlled air entrainment on the thermal and mechanical performance of dry-mixed wall mortar [29].

Based on this, DWAM was designed and prepared using DWRA combined with the super-insulating advantage of aerogel. The effects of P/A (30/70, 35/65, and 40/60), aerogel content (60 vol.%, 70 vol.%, 80 vol.%, 90 vol.%, and 100 vol.%), and AEA dosage (0.1 wt%, 0.2 wt%, 0.3 wt%, 0.4 wt%, and 0.5 wt%) on mortar performance were investigated. Microstructure and pore evolution were analyzed using scanning electron microscopy (SEM) and X-ray computed tomography (X-CT). This study aims to improve DW resource utilization, balance strength and thermal insulation, enhance material–building life matching, and provide a reference DWAM mix proportion.

## 2. Experimental

### 2.1. Materials

**Figure 1:**
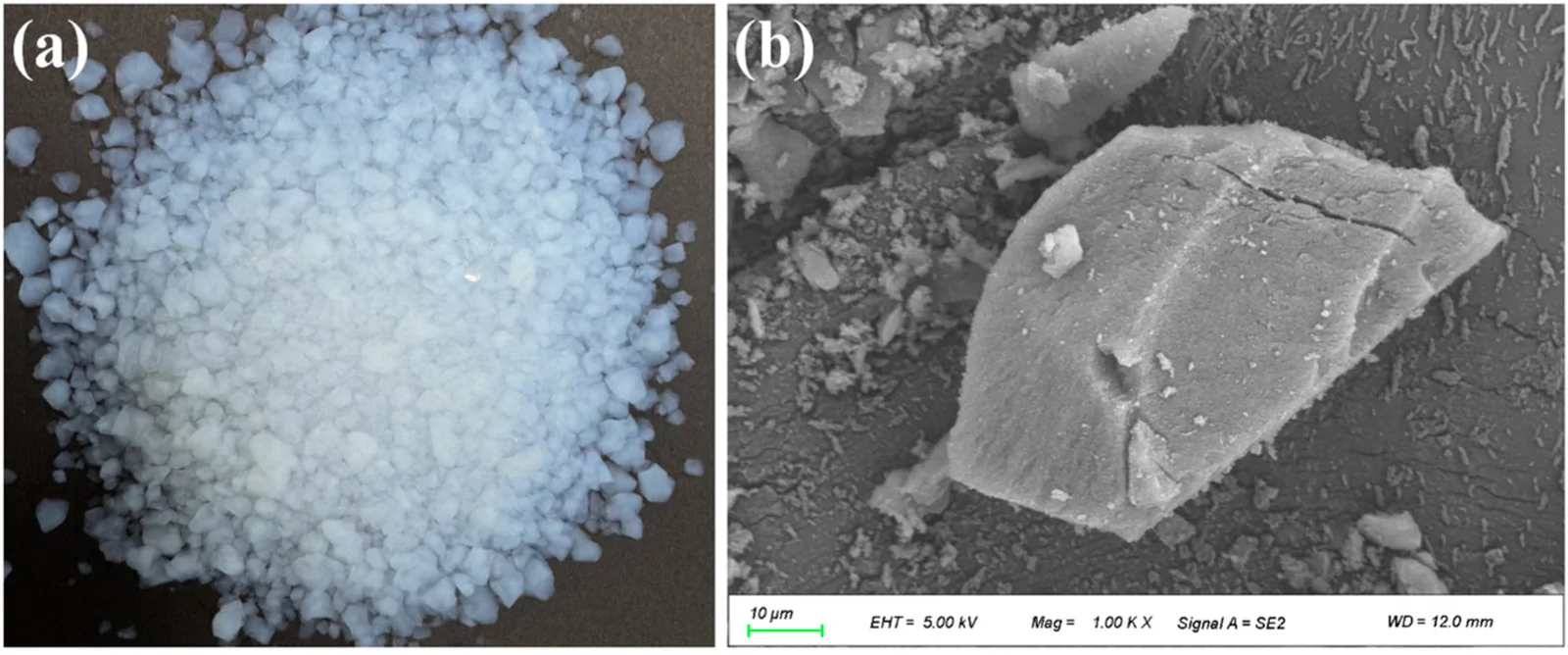
Silica aerogel: (**a**) macroscopic morphology; (**b**) SEM image.

Ordinary Portland cement (P.O 52.5) (supplied by Hubei Zhongxia Cement Co., Ltd., Hubei, Jingmen, China) was used as the primary binder to prepare mortar. S95 slag powder (supplied by Henan Yikerui New Materials Co., Ltd., Gongyi, China) and silica fume (supplied by Shandong Sanmei Silicon Materials Co., Ltd., Zibo, China) were selected as mineral admixtures to partially replace cement. Their main chemical compositions are given in Table 2. To prepare DWAM, hydrophobic silica aerogel particles were supplied by Guangdong Alison Hi-Tech Co., Ltd. (Jingmen, China) (Figure 1). Their main physical properties are listed in Table 3. KH-550 silane coupling agent from Dongguan Kangjin New Material Technology Co., Ltd. (Dongguan, China) served as the surface modifier for aerogel. DWRA was provided by Jiangsu Lvhe Environmental Technology Co., Ltd. (Changzhou, China). After removing impurities, the waste was crushed, ground, and sieved to obtain particles with a size range of 0–4.75 mm. The fineness modulus and apparent density of DWRA are 2.80 and 2150 kg/m^3^, respectively. The incorporation of multiple functional admixtures was intended to ensure adequate fresh state stability and mechanical integrity of the highly porous DWAM rather than solely to improve thermal insulation. Polycarboxylate superplasticizer (PCE) (provided by Jiangsu Subote New Materials Co., Ltd., Nanjing, China), with a water-reducing efficiency of approximately 40%, was used to improve the dispersion of cementitious particles and maintain workability without increasing the mixing water content. Hydroxypropyl methyl cellulose (HPMC) (provided by Shandong Heda Group Co., Ltd., Zibo, China) was incorporated to improve water retention and mixture cohesiveness and to mitigate segregation or floating of the low-density aerogel particles. Redispersible Latex Powder (RLP) (provided by Shandong Xindadi Industrial Group Co., Ltd., Jinan, China) was used to enhance cohesion and interfacial bonding and improve the flexural toughness of the mortar. Polypropylene (PP) fibers (provided by Changzhou Tianyi Engineering Fiber Co., Ltd., Changzhou, China) were incorporated to bridge microcracks and reduce the cracking susceptibility and brittleness associated with the porous matrix. The physical properties of PP fibers are listed in Table 4. AEA served a different function by introducing stable air voids into the cementitious matrix, thereby improving workability and reducing density and thermal conductivity. However, excessive air entrainment may adversely affect mechanical strength. In the basic mixtures, PCE and RLP were each maintained at 0.5 wt% of the binder, while HPMC and PP fibers were each maintained at 0.2 wt%. AEA was subsequently varied as an independent parameter to investigate its influence on the properties of DWAM.

### 2.2. Sample Preparation

This study adopted the volume method for mix design. Three paste-to-aggregate volume ratios, including 30/70, 35/65, and 40/60, were used and labeled DWAM1, DWAM2, and DWAM3, respectively. To obtain low-density DWAM with excellent thermal insulation, aerogel volume was set from 60% to 100% to replace DWRA by volume in mortar. Table 5 lists the specific mix proportions. After determining optimal P/A and aerogel content, the effect of AEA dosage on DWAM performance was investigated. Table 6 presents mix proportions for specimens with AEA.

The preparation procedure for DWAM was as follows. The mixing water was divided into two portions. One portion was premixed with PCE and set aside. The other portion was combined with a silane coupling agent to pretreat the aerogel particles. First, cementitious materials, DWRA, admixtures, and fibers were dry-mixed for 60 s. Then, the water containing PCE was gradually added and the mixture was further mixed for 90 s. Subsequently, the modified aerogel particles along with silane coupling agent treatment solution were added and mixing continued for an additional 120 s to obtain the fresh DWAM. The fresh mortar was cast in molds, allowed to rest for 24 h, and then demolded. All specimens were cured in a standard curing room at 20 ± 2 °C and relative humidity > 95% for 28 d. Three dimensions of specimens were prepared: 70.7 mm × 70.7 mm × 70.7 mm cubes for compressive strength and dry density tests, 40 mm × 40 mm × 160 mm prisms for flexural strength tests, and 300 mm × 300 mm × 30 mm slabs for thermal conductivity measurements.

### 2.3. Experimental Methods

#### 2.3.1. Workability Test

The workability of freshly mixed DWAM was characterized in terms of fluidity and consistency. The measurements were conducted immediately after completion of the mixing procedure. Fluidity was determined according to GB/T 2419-2005 [31]. Fresh mortar was placed into the standard flow mold, after which the mold was vertically removed and the flow table procedure specified in the standard was performed. The spread diameters measured in two mutually perpendicular directions were averaged to obtain the fluidity value. Consistency was measured using a mortar consistometer according to JGJ/T 70-2009 [32], and the penetration depth of the standard cone into the fresh mortar was recorded in millimeters.

#### 2.3.2. Dry Density Test

The dry density of hardened DWAM was determined according to GB/T 20473-2021 [33] using 70.7 mm × 70.7 mm × 70.7 mm cubic specimens. In this study, the density of hardened DWAM refers specifically to its oven-dry bulk density, hereafter termed “dry density”. After standard curing for 28 d at 20 ± 2 °C and a relative humidity greater than 95%, the specimens were dried in an oven at 105 ± 5 °C until a constant mass was reached. The external dimensions and oven-dry mass of each specimen were measured, and the dry density was calculated as the oven-dry mass divided by the external volume of the specimen. Three specimens were tested for each mixture, and the average value was reported.

#### 2.3.3. Mechanical Properties Test

The compressive and flexural strengths of DWAM were measured after 28 d of standard curing. Compressive strength was determined using 70.7 mm × 70.7 mm × 70.7 mm cubic specimens according to GB/T 5486 [34], whereas flexural strength was determined using 40 mm × 40 mm × 160 mm prismatic specimens according to GB/T 17671-2021 [35]. The specimens were loaded using a hydraulic servo universal testing machine until failure. The loading rates for compressive and flexural tests were 1.5 kN/s and 50 N/s, respectively. Three specimens were tested for each mixture, and the average strength was reported.

#### 2.3.4. Thermal Conductivity Test

The thermal conductivity of DWAM was measured using 300 mm × 300 mm × 30 mm slab specimens with a DD300R thermal conductivity tester (Tianjin Gangyuan Testing Instrument Factory, Tianjing, China) according to GB/T 10294-2008 [36] using the steady state guarded hot plate method. Prior to testing, the specimens were in dry state. The thermal conductivity was determined at a mean test temperature of 25 °C, with temperatures of 35 °C and 15 °C applied to the hot and cold surfaces, respectively.

#### 2.3.5. Microscopic Analysis

The microstructural morphology of DWAM was examined using a Tescan Mira 4 field emission scanning electron microscope (provided by TESCAN ORSAY HOLDING, a.s., Shanghai, China). Small representative fragments were collected from the interior of the hardened specimens and gold coating before SEM observation. X-ray computed tomography (nanoVoxel-4000, provided by Sanying Precision Instruments Co., Ltd., Tianjing, China) was performed to characterize the internal pore structure. The scanning voltage and current were 170 kV and 80 μA, respectively, and the reconstructed voxel resolution was approximately 17 μm. The acquired CT datasets were processed using Amira Avizo 2025 software with cropping, noise reduction, filtering, and image segmentation, followed by three-dimensional reconstruction. The reconstructed data were subsequently used to determine pore morphology, equivalent pore size distribution, spatial distribution, and overall porosity.

## 3. Results and Discussion

### 3.1. Effect of Paste-to-Aggregate Ratio and Aerogel Content on the Properties of DWAM

#### 3.1.1. Workability

**Figure 2:**
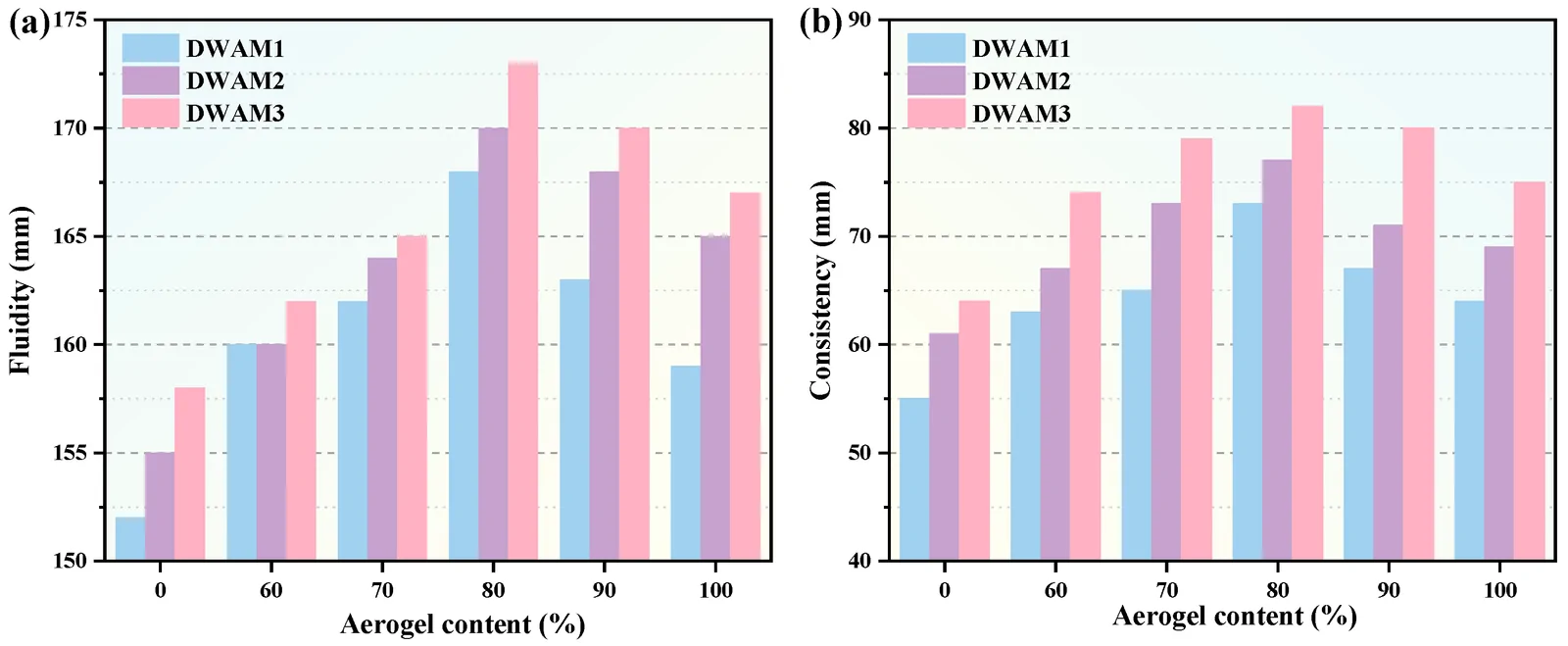
(**a**) Fluidity and (**b**) consistency of DWAM with different P/A and aerogel content.

The workability of the mortar was evaluated using consistency and fluidity. Figure 2 presents the variations of these two indicators for DWAM under different P/A and aerogel content. Both fluidity and consistency increased with the P/A. A lower ratio led to higher slurry viscosity and thus poorer workability, whereas a higher ratio reduced viscosity and improved fluidity and consistency. At an aerogel content of 100%, compared to DWAM1, the fluidity of DWAM2 and DWAM3 increased by 3.7% and 5.0%, and their consistency rose by 7.8% and 17.2%, respectively.

Aerogel content exerts a more pronounced effect on workability than the P/A. As aerogel content increased, the fluidity of mortars with all three ratios improved significantly, peaking at 80% aerogel. At this optimum, the fluidity of DWAM1 was 10.5% higher than that of the mortar without aerogel (Figure 2a). Consistency followed a similar trend (Figure 2b). When the aerogel content in DWAM3 reached 80%, consistency attained 82 mm. This enhancement was attributed to the reduced proportion of rough surfaced DWRA, which lowered internal flow resistance. However, beyond 80% aerogel content, both fluidity and consistency began to decline. Owing to the large specific surface area of aerogel, higher aerogel content required more cement paste to fully encapsulate the particles for improved flowability under a fixed P/A, and this eventually deteriorated workability.

Thus, aerogel content significantly influenced mortar workability, with the optimum at 80% aerogel. According to relevant standards [37], the consistency of masonry mortar should range from 60 mm to 90 mm to ensure adequate spreadability and constructability. At 80% aerogel content, DWAM3 achieved a consistency of 82 mm, fully meeting the standard. Moreover, its excellent fluidity enhanced construction efficiency and reduced the risk of crack propagation.

#### 3.1.2. Dry Density

**Figure 3:**
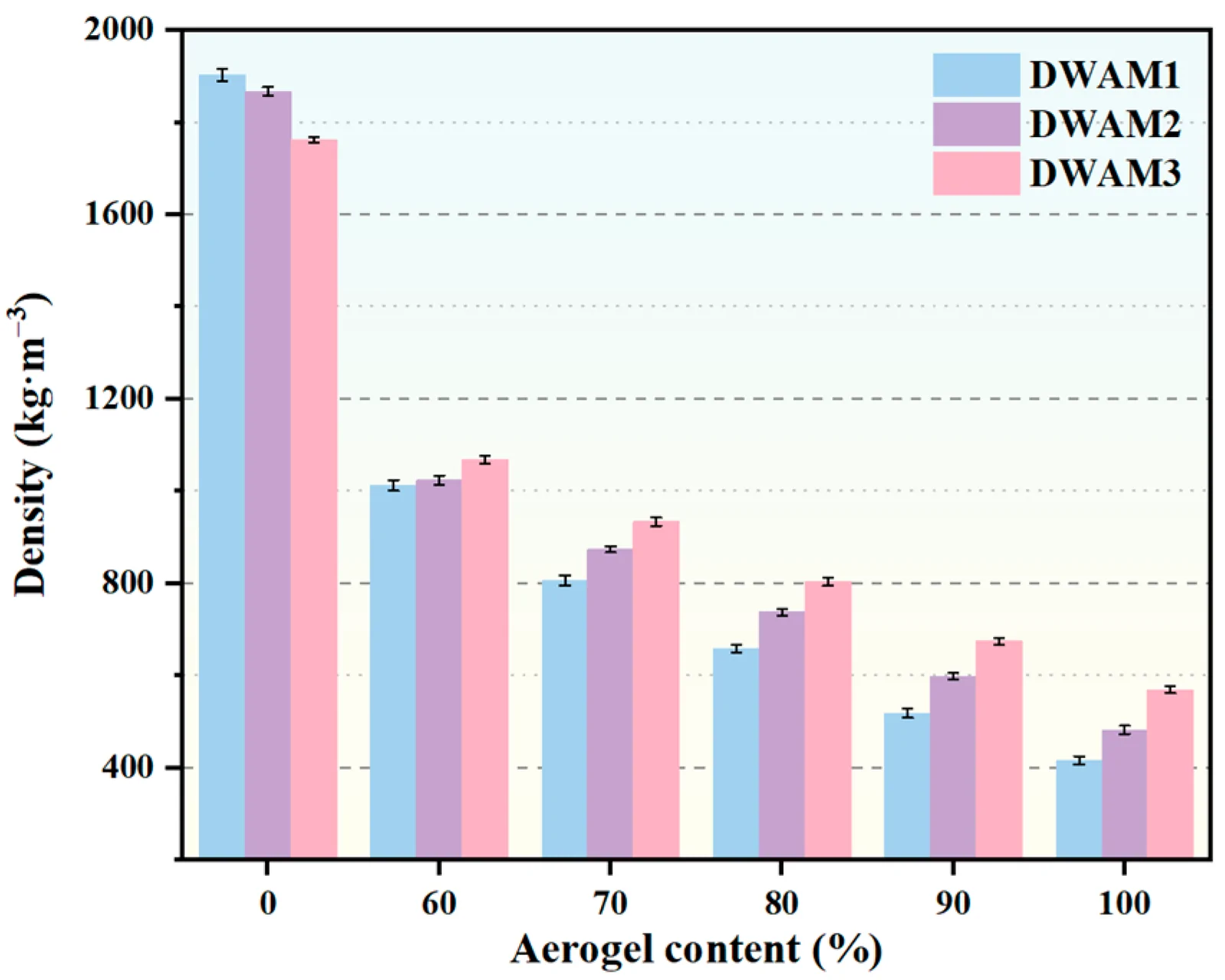
Dry density of DWAM with different P/A and aerogel content.

Under different P/A conditions, the effect of aerogel content on the density of DWAM is shown in Figure 3. The density of silica aerogel particles is 100 kg/m^3^, which is much smaller than that of DWRA. Therefore, as aerogel particles gradually replaced the DWRA by volume, the overall density of DWAM decreased. For a given aerogel content, the most significant reduction was observed for DWAM1. At an aerogel content of 60%, its density reached 1012.4 kg/m^3^. When the aerogel content increased to 100%, the density further dropped to 416.3 kg/m^3^. On average, every 10% increase in aerogel content led to an approximately 14.7% decrease in density. The results indicated that a smaller P/A and a higher aerogel replacement ratio both resulted in a lower mortar density.

#### 3.1.3. Mechanical Properties

**Figure 4:**
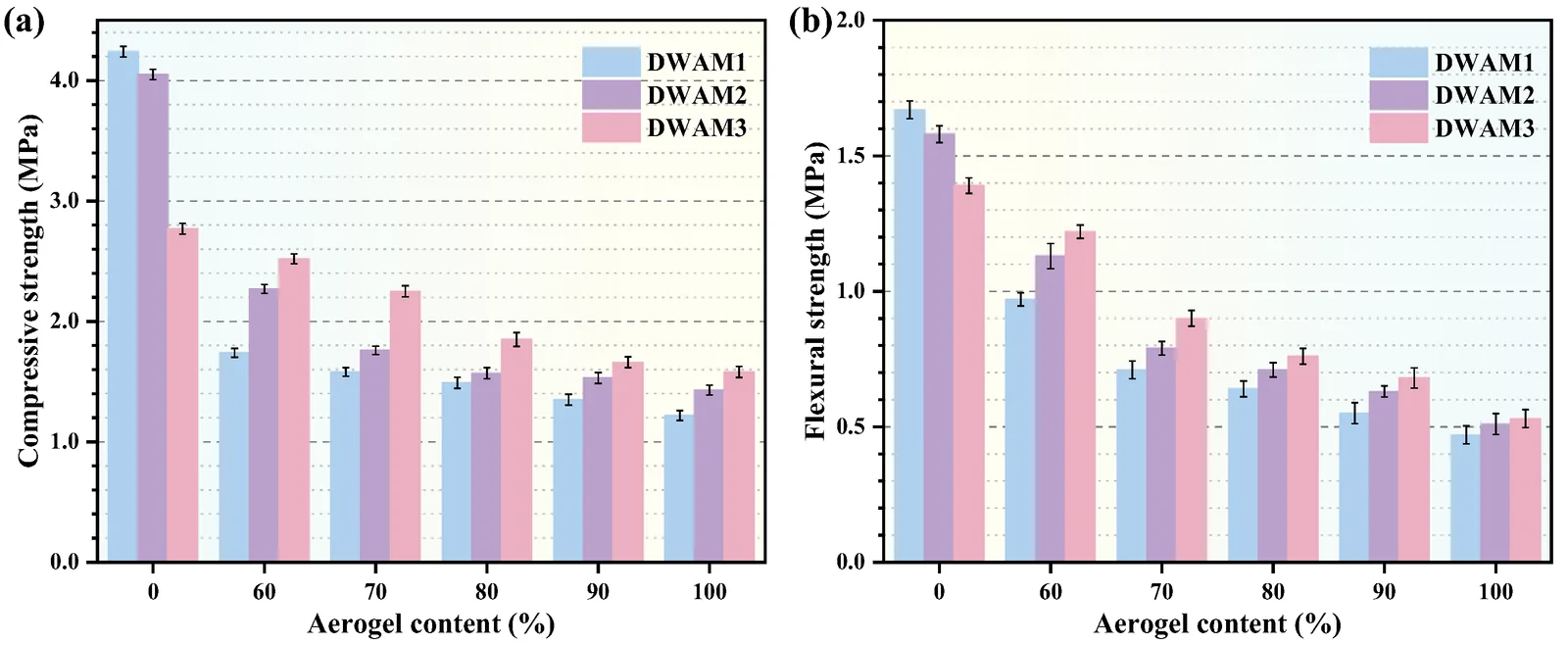
(**a**) Compressive strength and (**b**) flexural strength of DWAM with varying paste-to-aggregate ratio and aerogel content.

Figure 4 presents the effects of P/A and aerogel content on the compressive and flexural strengths of DWAM. As the aerogel content increased from 60% to 100%, the strength of the mortar gradually declined. Among these mixtures, DWAM3 exhibited the most pronounced reduction. Its compressive strength decreased from 2.52 MPa to 1.58 MPa with a drop of 37.3%, while its flexural strength fell from 1.22 MPa to 0.53 MPa, corresponding to a 56.6% decrease. With an average increase of 10% aerogel, the compressive and flexural strengths decreased by 9.3% and 14.1%, respectively.

In contrast, DWAM1 showed the smallest decline, with compressive and flexural strengths decreasing by 7.5% and 12.9% on average, respectively, for each 10% increase in aerogel content. The significant deterioration in the mechanical properties of aerogel mortar could be attributed to the increasing volume fraction of aerogel, which inherently possessed very poor mechanical properties. When a large amount of aerogel was incorporated into the mortar, the porosity increased, thereby reducing the strength. Notably, DWAM3 demonstrated a better bonding effect between the paste and aggregates, resulting in a more uniform internal pore structure. Consequently, compared with the other two groups, DWAM3 achieved higher compressive and flexural strengths. However, when the P/A was less than 40/60, the addition of a large amount of aerogel during mixing partially damaged the aerogel particles. Moreover, the insufficient amount of paste failed to completely wrap the aggregates, resulting in uneven distribution of aggregate within the mortar and weakened overall performance.

#### 3.1.4. Thermal Conductivity

**Figure 5:**
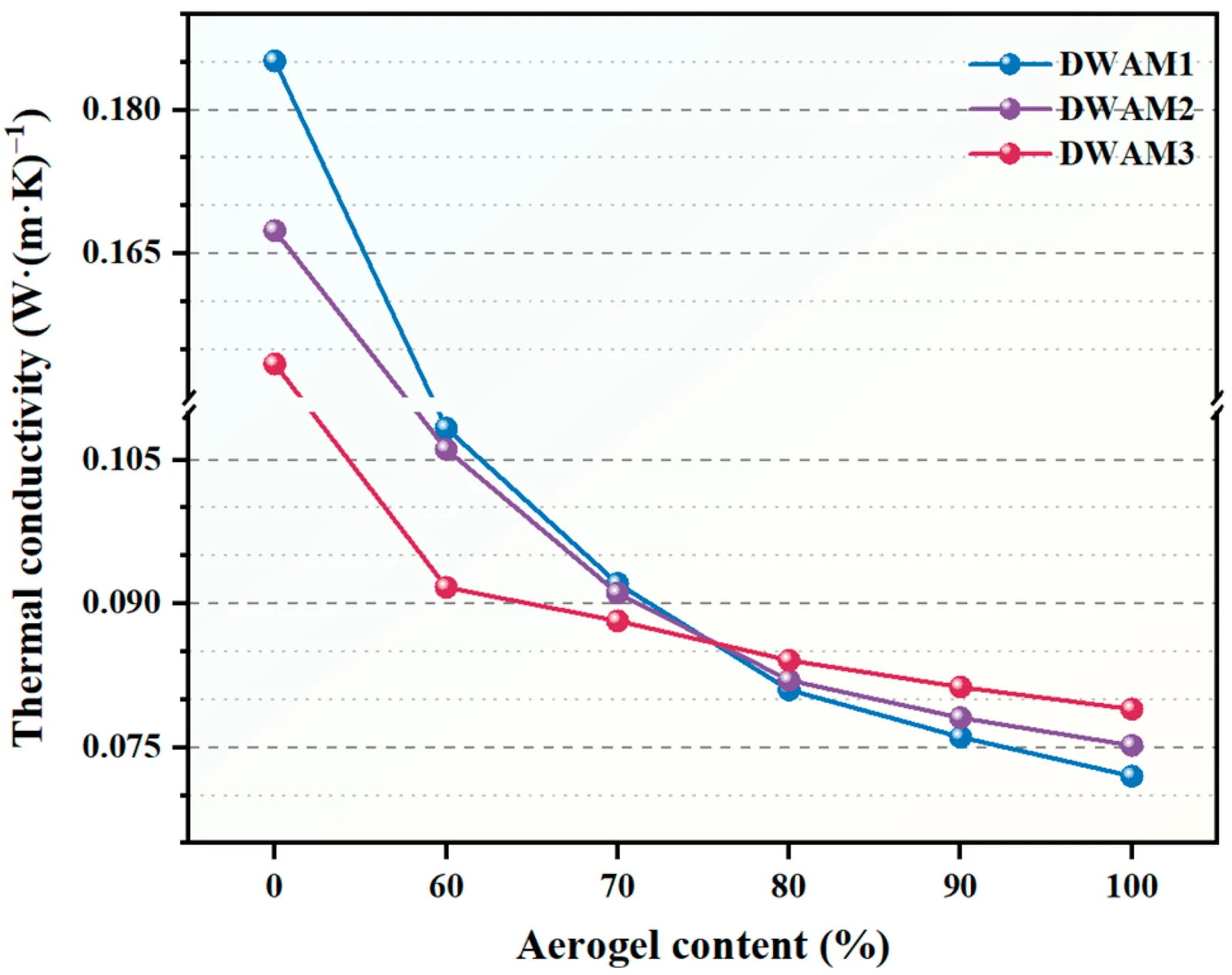
Thermal conductivity of DWAM with varying paste-to-aggregate ratio and aerogel content.

Figure 5 illustrates the relationship between the thermal conductivity of DWAM and both the P/A and the aerogel content. As the aerogel volume fraction increased, the thermal conductivity of DWAM with different P/A values exhibited varying degrees of reduction. Specifically, when the aerogel content ranged from 60% to 100%, DWAM1 and DWAM2 showed a more rapid decline in thermal conductivity compared to DWAM3. Among all mixtures, DWAM1 demonstrated the largest reduction. Its thermal conductivity decreased from 0.1083 W/(m·K) at 60% aerogel content to 0.0720 W/(m·K) at 100% aerogel content. Every 10% increase in aerogel content resulted in an average 8.3% decrease in the thermal conductivity. When the aerogel content exceeded 70%, the thermal conductivities of the mortars ranked as DWAM1 < DWAM2 < DWAM3. This suggested that the thermal conductivity of the mortar tended to decrease with a reduction in paste content and a corresponding increase in aggregate volume.

### 3.2. Effect of AEA Dosage on the Properties of DWAM

AEA can introduce tiny, stable air bubbles into the mortar mixture. This not only improves workability but also creates air voids that enhance thermal insulation performance of the mortar. Based on the findings presented above, and taking into account both thermal and mechanical properties, this section explored the effect of AEA dosage (0.1 wt% to 0.5 wt%) on the properties of DWAM, with the P/A fixed at 40/60 and the aerogel content at 80%.

#### 3.2.1. Workability

**Figure 6:**
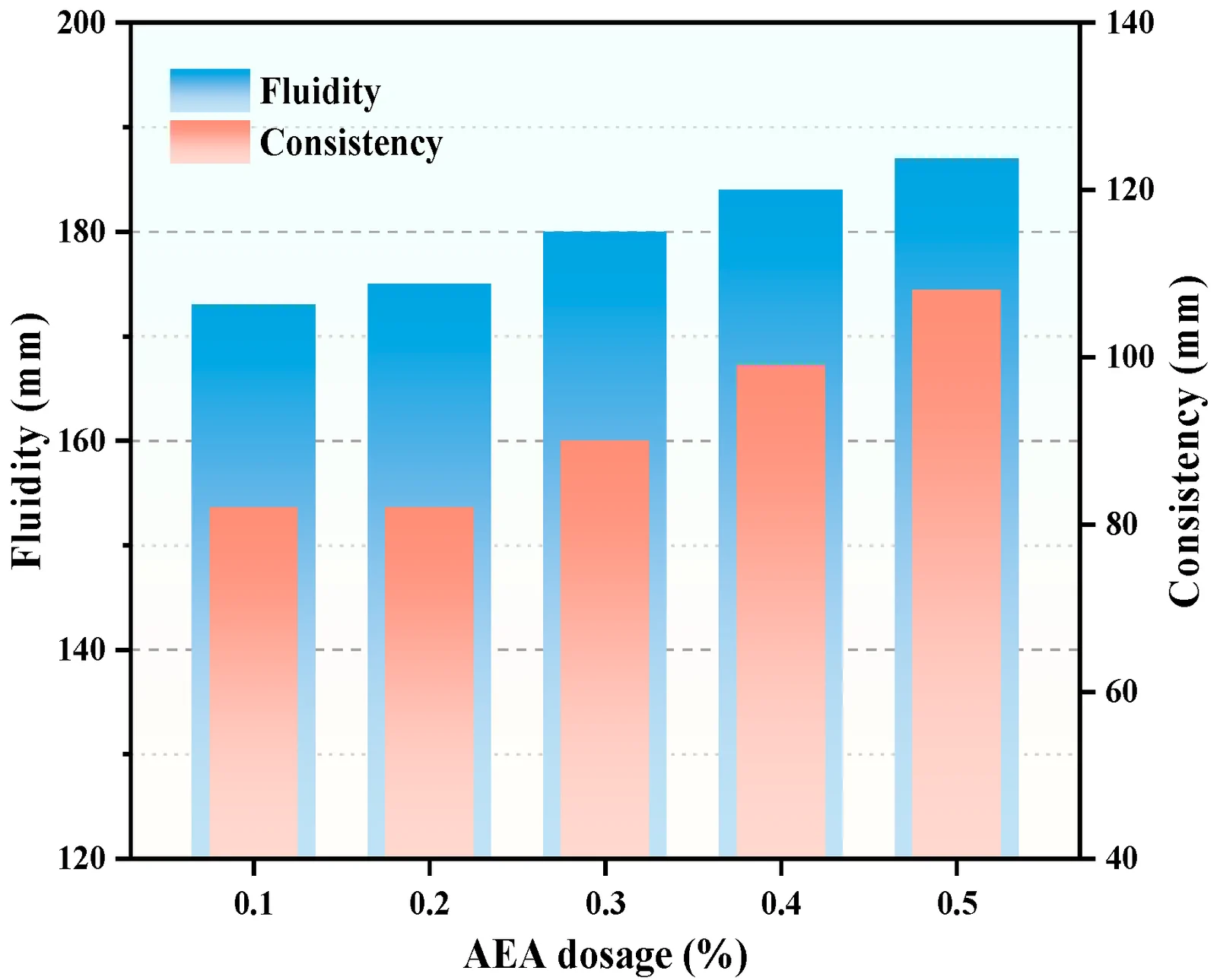
Effect of AEA dosage on fluidity and consistency of DWAM.

Figure 6 displays the variation trend of DWAM as the AEA dosage increases. Both the fluidity and consistency values of the DWAM exhibited a linear increase with the rising dosage of the AEA. At 0.2% AEA, the fluidity and consistency were 175 mm and 82 mm, respectively. When the AEA dosage was increased to 0.5%, the fluidity and consistency rose to 187 mm and 108 mm, respectively. On average, each 0.1% increase in AEA led to a 2.1% increase in fluidity and a 10.6% increase in consistency. AEA is an interfacial active agent whose activity primarily occurs at the gas–liquid interface [38]. As the AEA dosage continued to increase, the interfacial activity at the gas–liquid interface of the mortar also increased, introducing a large number of fine bubbles into the mortar slurry. These bubbles were arranged in a regular and stable manner, and their internal presence gradually reduced the internal flow resistance of the mortar, thereby continuously improving the fluidity and consistency of the aerogel mortar.

#### 3.2.2. Dry Density

**Figure 7:**
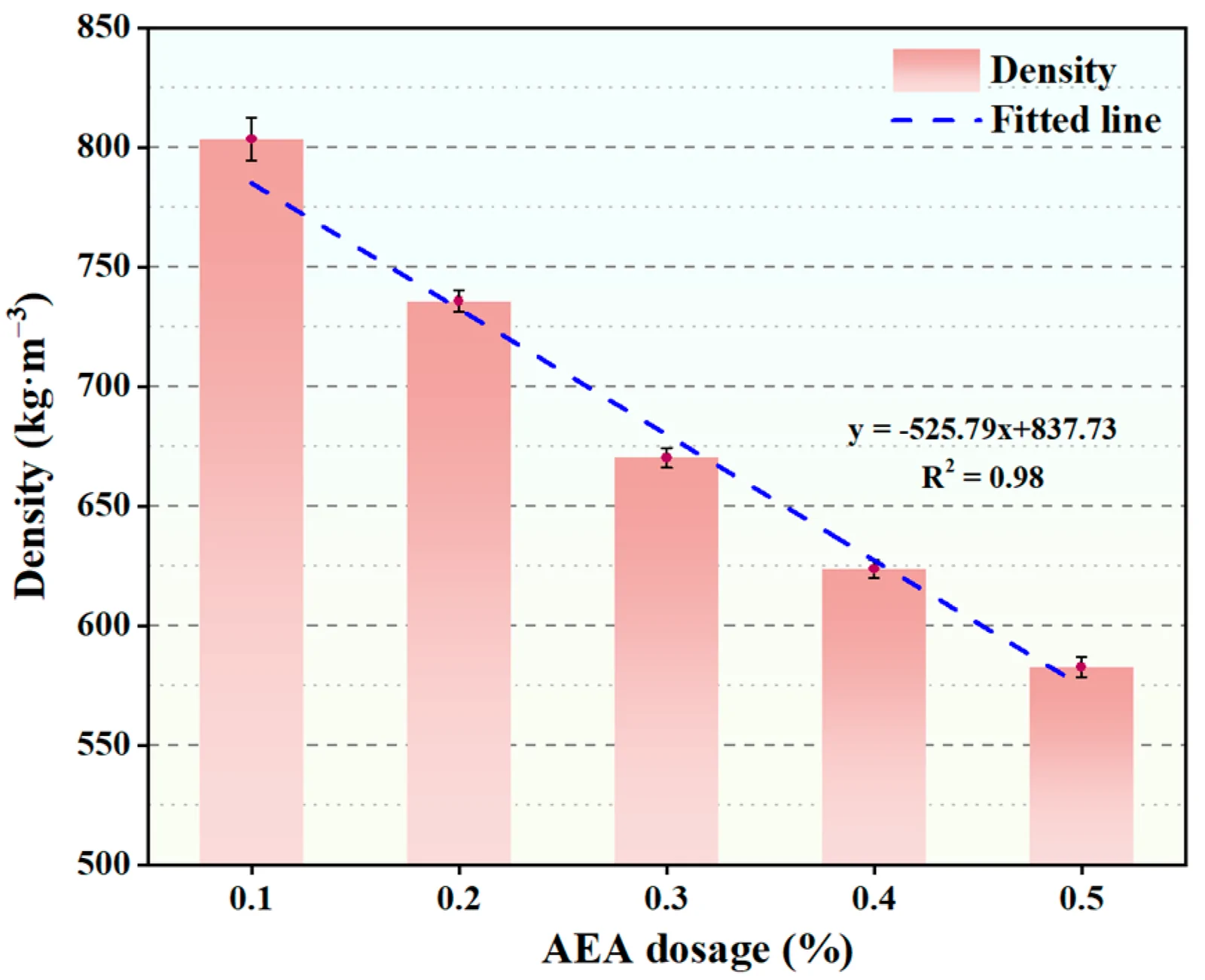
Effect of AEA dosage on dry density of DWAM.

The effect of AEA dosage on the density of DWAM is shown in Figure 7. The density of DWAM showed a significant downward trend as the dosage of AEA increased. When increasing the AEA content from 0.1% to 0.5%, the dry density was reduced from 803.6 kg/m^3^ to 582.7 kg/m^3^. This reduction was attributed to the incorporation of substantial stable air bubbles into the mortar, which raised the air volume and consequently reduced the material mass per unit volume. Furthermore, as the AEA dosage gradually increased, more and more pores formed within the mortar, leading to a further decrease in the density of DWAM. A linear fit was applied to the data, revealing a strong negative linear correlation between AEA dosage and density (R^2^ = 0.98). This indicates that as the AEA content increases, the density decreases in a nearly perfectly linear manner, without significant deviation. The consistent downward trend suggests that each increase in AEA introduces a proportional amount of air voids, steadily reducing the unit mass of DWAM.

#### 3.2.3. Mechanical Properties

**Figure 8:**
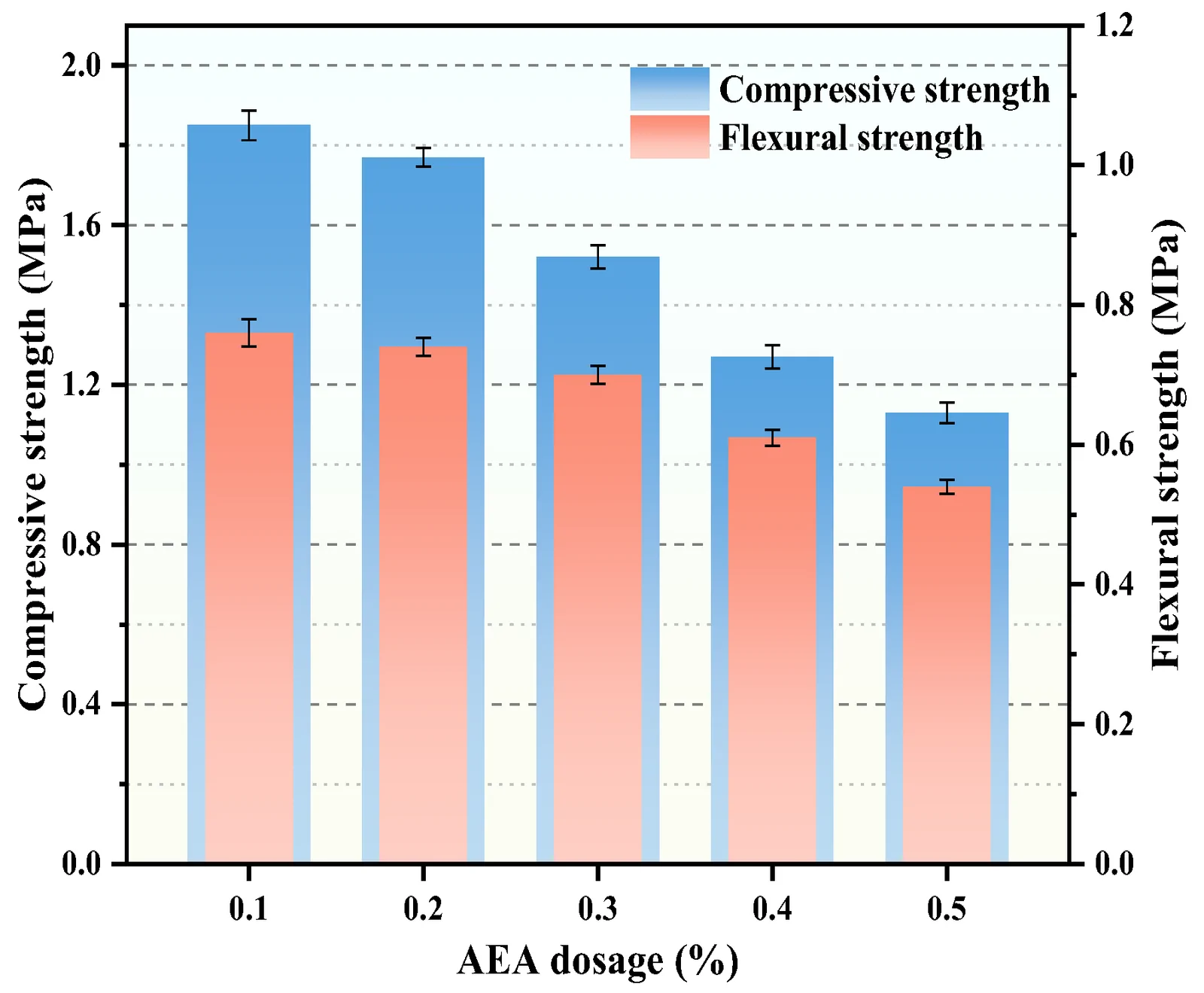
Effect of AEA dosage on mechanical properties of DWAM.

Figure 8 shows the evolution of the mechanical properties of DWAM with different dosages of AEA. With increasing AEA dosage, both the 28-d compressive and flexural strengths of DWAM exhibited a downward trend. The compressive strength decreased from 1.83 MPa at 0.1% AEA to 1.13 MPa at 0.5%, representing a reduction of 36.2%. The flexural strength decreased from 0.74 MPa at 0.1% AEA to 0.54 MPa at 0.5%, with a reduction of 27.0%. This phenomenon was due to the increase in air volume within the mortar, which also enlarged the volume of the cement paste, expanded the gap between the aggregates, and consequently reduced the compactness and uniformity of DWAM.

At low AEA dosages (below 0.3%), the loss rates of both compressive and flexural strength were relatively low, especially for flexural strength. As the AEA dosage increased, more air bubbles were introduced, leading to a significant decline in both compressive and flexural strengths. The results indicated that the addition of AEA had a greater impact on compressive strength than on flexural strength.

#### 3.2.4. Thermal Conductivity

**Figure 9:**
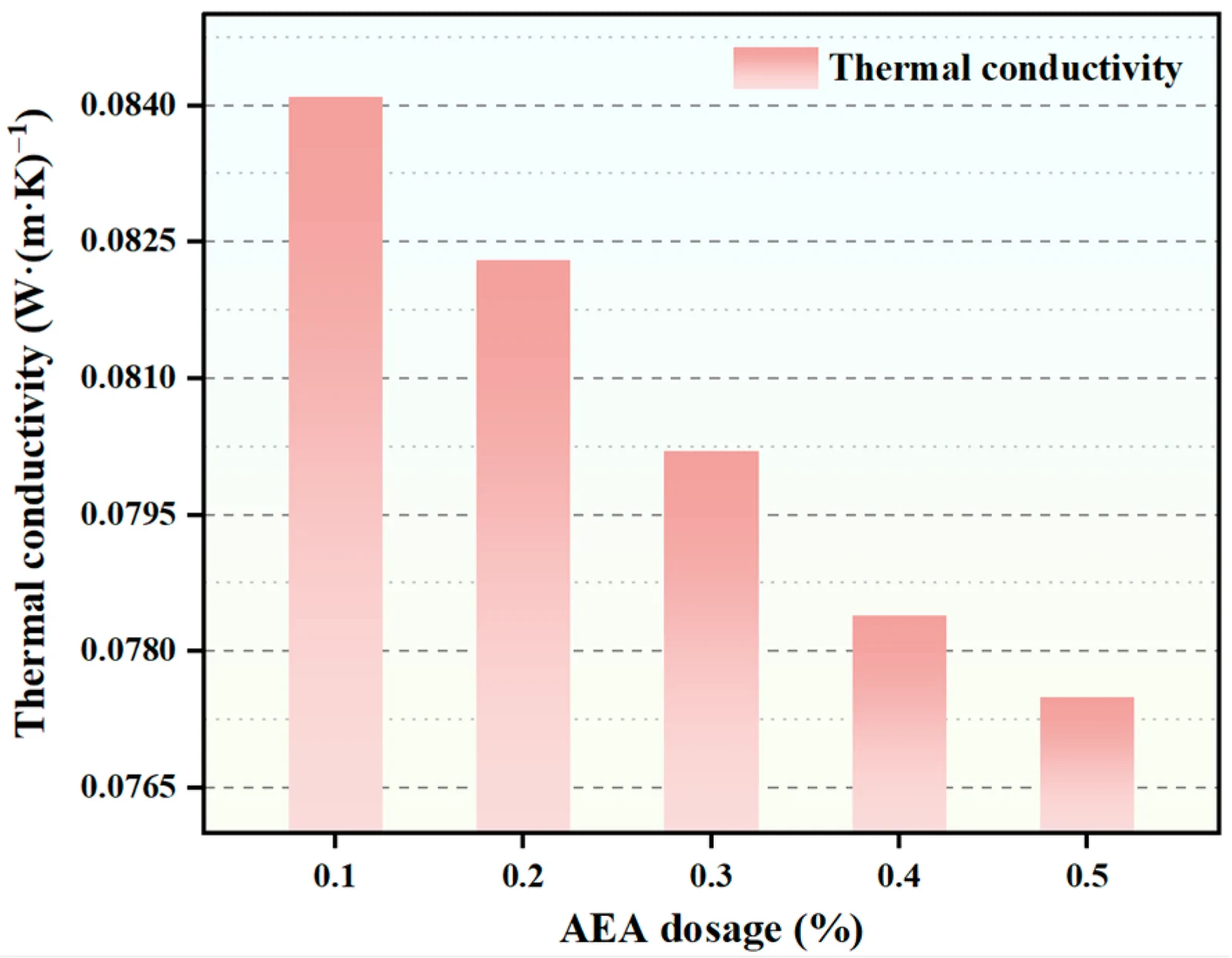
Thermal conductivity variation of DWAM with different AEA dosage.

The results of AEA dosage affecting the thermal conductivity of DWAM are shown in Figure 9. Increasing the AEA dosage from 0.2% to 0.5%, the thermal conductivity was reduced from 0.0823 W/(m·K) to 0.0775 W/(m·K), corresponding to an average decrease of 5.8% for each 0.1% increase in AEA dosage. In addition, the incorporation of AEA introduced a large number of spherical closed bubbles during the mixing process of DWAM. These bubbles varied in pore size, and the porosity and air content of DWAM were also improved. Since the heat capacity of the gas is much lower than that of the solid medium, the heat transfer rate within the aerogel mortar is reduced, thereby improving the thermal insulation performance.

### 3.3. SEM Analysis

**Figure 10:**
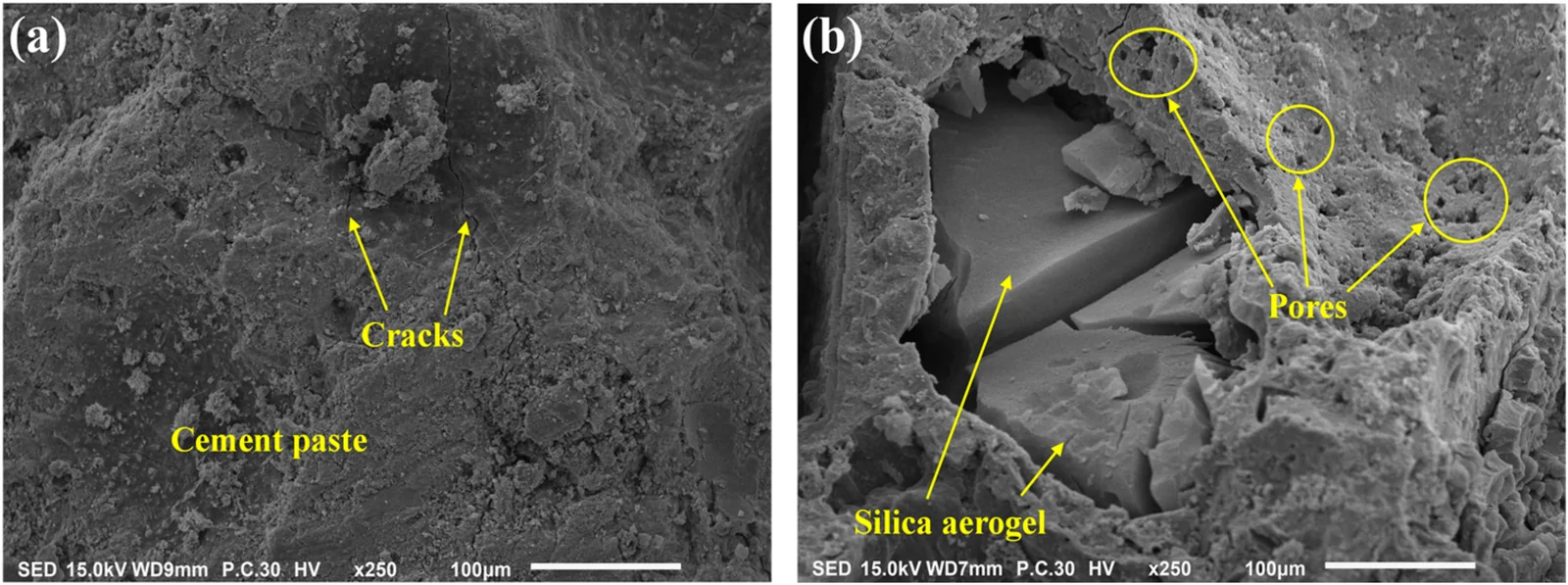
SEM images of DWAM: (**a**) DWAM3 without aerogel; (**b**) DWAM3-A3.

Figure 10a and Figure 10b show the SEM images of DWAM3 (without aerogel) and DWAM3-A3, respectively. As observed in Figure 10a, in the absence of aerogel, the mortar exhibited fewer micropores and microcracks, and the matrix was denser. This microscopically explained why DWAM without aerogel had higher dry density and better mechanical properties.

Figure 10b reveals that after aerogel incorporation, the internal porosity of DWAM increased. Due to the inherent hydrophobicity of aerogel, gaps persisted between the aerogel particles and the cement matrix, even after modification with a silane coupling agent. During the preparation of DWAM, aerogel particles, which had extremely low strength, were easily broken into smaller fragments and embedded into the cement paste upon mixing. However, owing to its extremely high porosity and specific surface area, silica aerogel remained capable of altering the heat transfer paths inside the mortar, even after being broken down into fine particles during mixing. Meanwhile, the surface of aerogel encased in cement paste remained clean, smooth and stable after cement hydration, indicating that silica aerogel was not affected by the alkaline environment generated during hydration. This also explained why the thermal insulation performance of DWAM was significantly influenced by the aerogel dosage. Furthermore, in the DWAM3-A3 sample, the increased AEA dosage introduced a large number of closed micropores into the cement paste (Figure 10b). These micropores reduced free air circulation, weakened heat transfer within DWAM, and further optimized the thermal insulation performance of the mortar. This was further confirmed by the following X-CT results.

### 3.4. X-CT Analysis

**Figure 11:**
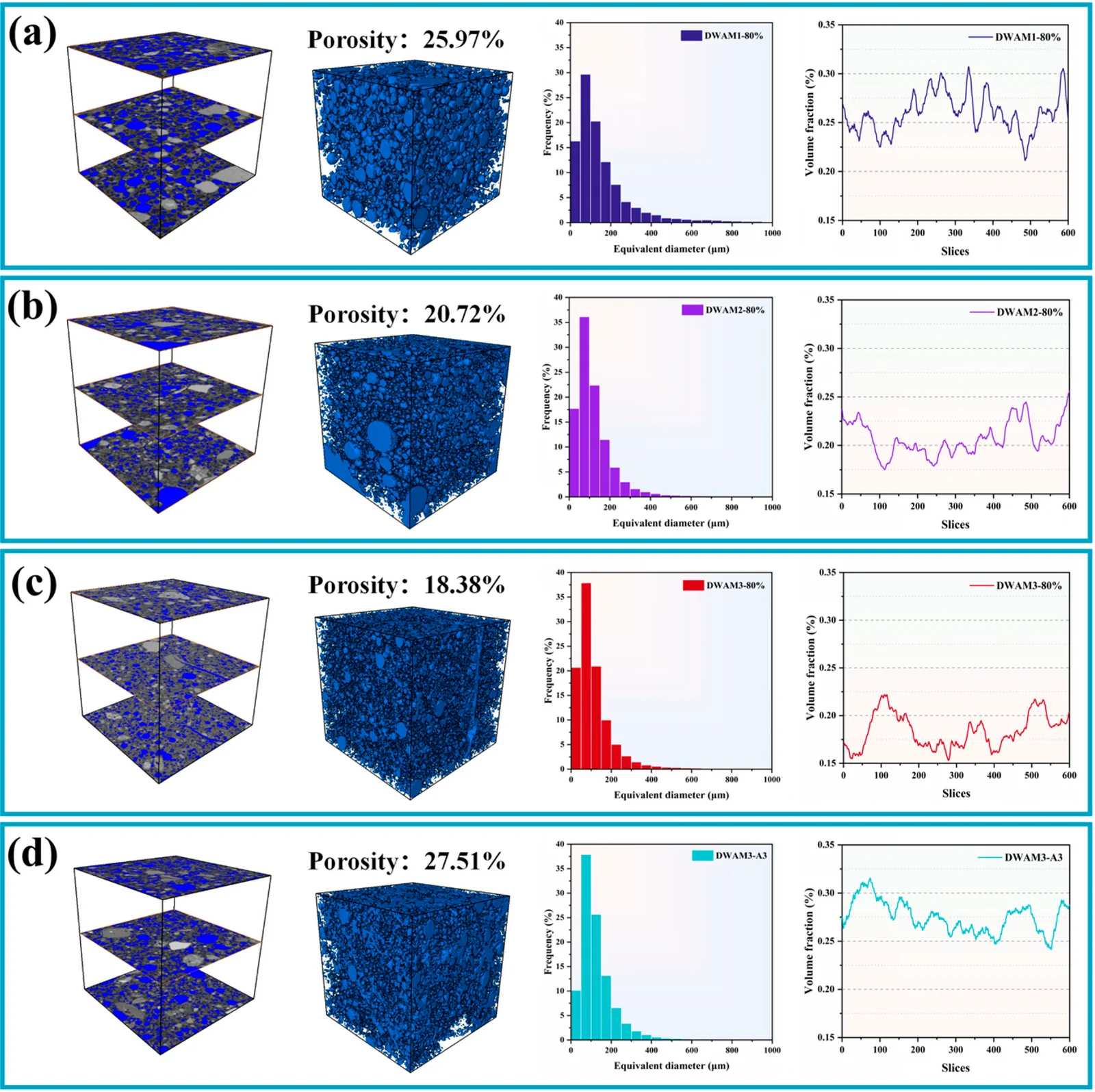
Pore slices, 3D pore rendering, frequency distribution of pore equivalent diameter, and layer-by-layer porosity of DWAM: (**a**) DWAM1-80%; (**b**) DWAM2-80%; (**c**) DWAM3-80%; (**d**) DWAM3-A3.

Figure 11 presents the pore slices, 3D pore rendering, frequency distribution of equivalent pore diameter, and layer-by-layer porosity of DWAM specimens. The X-CT results indicated that the thermal conductivity of DWAM was not determined solely by the aerogel dosage, but was co-regulated by the size distribution, morphology, and connectivity of internal pores. The overall porosities of DWAM1-80%, DWAM2-80%, DWAM3-80%, and DWAM3-A3 were 25.97%, 20.72%, 18.38%, and 27.51%, respectively, with corresponding thermal conductivities of 0.0810, 0.0820, 0.0841, and 0.0784 W/(m·K). At a fixed aerogel content, as the P/A increased from 30/70 to 40/60, the matrix compactness improved, overall porosity decreased, and thermal conductivity increased. This indicated that the increased heat conduction path through the mortar skeleton weakened the thermal insulation contribution of air voids.

The pore size frequency distribution showed that DWAM3-80% exhibited the highest relative frequency in the pore size range of 50–100 μm and the lowest proportion of large pores >200 μm, explaining its lowest porosity and high compressive strength of 1.85 MPa. In contrast, for specimen DWAM3-A3 with an AEA dosage of 0.4%, the overall porosity increased to 27.51%, pores were concentrated in the 50–200 μm range, and the proportion of pores larger than 200 μm increased slightly. These closed air voids effectively interrupted solid heat transfer and significantly improved thermal insulation performance by leveraging the low thermal conductivity of air. From the layer-by-layer porosity distribution in Figure 11d, DWAM3-A3 also exhibited the best pore distribution uniformity, which facilitated homogeneous heat flow conduction in all directions and avoided localized thermal bridging effects. However, the emergence of numerous pore bubbles inevitably introduced a small number of interconnected pores and coarse pores, leading to a reduction in compressive strength.

### 3.5. Discussion

The results from workability, mechanical tests, thermal conductivity measurements, and microscopic analyses (SEM and X-CT) consistently reveal a pore structure performance mechanism governing DWAM. Increasing the P/A improves matrix compactness and workability, whereas aerogel incorporation reduces dry density and thermal conductivity by introducing porous particles that disrupt solid heat transfer. However, beyond 80 vol.% aerogel, the high specific surface area of aerogel demands more paste for encapsulation, leading to deteriorated workability and strength loss. The AEA further introduces closed micropores, which effectively lower thermal conductivity (from 0.0823 W/(m·K) to 0.0775 W/(m·K) as AEA increases from 0.2 wt% to 0.5 wt%) but inevitably cause stress concentration and strength reduction, as confirmed by X-CT. Notably, X-CT reveals that DWAM3-A3 exhibits the most uniform pore distribution, which facilitates homogeneous heat flow and avoids localized thermal bridging, explaining its superior thermal insulation despite a moderate strength loss. The optimal mixture (40/60 of P/A, 80 vol.% of aerogel, and 0.4 wt% of AEA) achieves a balanced performance, satisfying the requirements of GB/T 20473-2021 [33] (thermal conductivity ≤0.085 W/(m·K) and compressive strength ≥1.0 MPa), with a measured thermal conductivity of 0.0784 W/(m·K) and compressive strength of 1.27 MPa. This demonstrates that rational design of pore structure can reconcile the trade-off between thermal insulation and mechanical strength in sustainable aerogel mortars.

From an environmental and economic perspective, the proposed DWAM presents both potential advantages and challenges. The utilization of DWRA provides a pathway for diverting decoration waste from landfill and reducing the demand for virgin natural aggregate. In addition, the incorporation of slag and silica fume as supplementary cementitious materials partially reduces the Portland cement fraction while providing a value-added route for industrial byproducts. The low thermal conductivity achieved by DWAM may also contribute to reduced operational energy demand when the material is applied as a building insulation layer. However, silica aerogel remains a relatively high-value constituent and is expected to be one of the principal contributors to the material cost and embodied environmental impact of DWAM. In this respect, introducing closed air voids through a small dosage of AEA provides an additional mechanism for reducing thermal conductivity without relying solely on further increases in aerogel content. The overall feasibility of DWAM will nevertheless depend on local decoration waste processing and transportation, aerogel production and price, surface modification, service life, and construction requirements. Therefore, although the present results demonstrate the technical potential of DWAM, a quantitative life cycle assessment and life cycle cost analysis are required in future work to establish its environmental and economic competitiveness at engineering scale.

## 4. Conclusions

This study systematically investigated the effects of P/A, silica aerogel content, and AEA dosage on the workability, mechanical properties, thermal conductivity, and microstructure of DWAM. Based on the experimental results and microscopic analyses, the following conclusions were drawn:

(1) Decreasing P/A and increasing aerogel content reduced the dry density, mechanical strength, and thermal conductivity of DWAM, while workability peaked at 80 vol.% aerogel. Beyond this optimum, the high specific surface area of aerogel requires more paste for encapsulation, increasing flow resistance and deteriorating workability.
(2) DWAM reached the ideal workability at a fixed P/A of 40/60, aerogel content of 80 vol.%, and AEA dosage of 0.1%. The fluidity and consistency were 173 mm and 82 mm, respectively, and the dry density reached 803.6 kg/m^3^, which met the engineering conditions. DWAM3-80% also exhibited a compressive strength of 1.85 MPa, a flexural strength of 0.76 MPa, and a thermal conductivity of 0.0841 W/(m·K). The uniform and finely distributed pore structure observed at the microscale provides a microstructural explanation for this synergistic effect. The air-filled pores increase thermal resistance by reducing the continuity of heat conduction paths through the solid cementitious skeleton. Meanwhile, the relatively homogeneous pore distribution suppresses the formation of excessively large and interconnected voids.
(3) Increasing AEA dosage from 0.2 wt% to 0.5 wt% progressively enhanced workability and thermal insulation but reduced dry density and mechanical strength. SEM and X-CT revealed that closed micropores introduced by AEA effectively interrupt solid heat transfer, while excessive porosity causes stress concentration and strength loss.
(4) The optimal mixture (40/60 of P/A, 80 vol.% of aerogel, and 0.4 wt% of AEA) achieved a dry density of 623.8 kg/m^3^, compressive strength of 1.27 MPa, flexural strength of 0.61 MPa, and thermal conductivity of 0.0784 W/(m·K). This formulation offers a sustainable insulating mortar with balanced properties suitable for building applications.

## Figures and Tables

**Table 1:** Comparison of the thermal properties of representative cementitious mortars containing waste-derived and lightweight materials.

Reference	Mortar Type	Waste/Lightweight Material	Material Proportion	Density (kg/m^3^)	Thermal Conductivity, λ (W/(m·K))
Koksal et al. (2020) [25]	Thermal insulation mortar	Expanded vermiculite + waste EPS	Aggregate/cement = 3–7 by volume; different EV/EPS ratios	393–946	0.09
Nasry et al. (2021) [27]	Cement mortar	Waste glass powder (WGP)	60 wt% cement replacement	1830	0.86
Marof and Šiller (2025) [28]	Thermal-insulating cement mortar	Recycled PET + silica aerogel	3 vol.% PET + 7 vol.% silica aerogel	1752	0.76
Di Maio et al. (2018) [30]	Cementitious lightweight mortar	Foamed end-of-waste plastic aggregate	50 vol.% replacement of natural sand	1374	1.266

**Table 2:** Chemical composition of cementitious materials (%).

Composition	CaO	SiO_2_	Al_2_O_3_	Fe_2_O_3_	MgO	SO_3_	K_2_O	TiO_2_	MnO	Others
Cement	63.21	21.08	6.37	3.85	1.68	1.17	0.63	0.24	0.15	1.62
Slag	53.96	21.56	12.46	0.74	7.98	1.03	0.58	-	-	1.69
Silica fume	1.21	95.20	0.07	-	0.08	-	0.01	-	-	3.43

**Table 3:** Physical properties of silica aerogel.

Particle Size (mm)	Density (kg/m^3^)	Pore Size (nm)	Specific Surface Area (m^2^/g)	Thermal Conductivity (W/m·K)	Porosity (%)	Contact Angle (°)
0.1–2	80–150	≤70	>500	0.012–0.023	>90	>140

**Table 4:** Physical properties of PP.

Density (g/cm^3^)	Diameter (mm)	Tensile Strength (MPa)	Elastic Modulus (MPa)	Elongation at Break (%)
0.98	0.02	481	4869	21

**Table 5:** Mix proportion of DWAM (kg/m^3^).

Series	Aerogel Volume	Cement	Slag	Silica Fume	DWRA	Aerogel	Water	KH-550	PCE	AEA	RLP	HPMC	PP
DWAM1	0	225	60	15	1505.0	0	105	2.63	1.50	0.30	1.50	0.60	0.60
	60%	225	60	15	602.0	42.0	105	2.63	1.50	0.30	1.50	0.60	0.60
	70%	225	60	15	451.5	49.0	105	2.63	1.50	0.30	1.50	0.60	0.60
	80%	225	60	15	301.0	56.0	105	2.63	1.50	0.30	1.50	0.60	0.60
	90%	225	60	15	150.5	63.0	105	2.63	1.50	0.30	1.50	0.60	0.60
	100%	225	60	15	0	70.0	105	2.63	1.50	0.30	1.50	0.60	0.60
DWAM2	0	274	73	18	1397.5	0	128	3.20	1.83	0.37	1.83	0.73	0.73
	60%	274	73	18	559.0	39.0	128	3.20	1.83	0.37	1.83	0.73	0.73
	70%	274	73	18	419.3	45.5	128	3.20	1.83	0.37	1.83	0.73	0.73
	80%	274	73	18	279.5	52.0	128	3.20	1.83	0.37	1.83	0.73	0.73
	90%	274	73	18	139.8	58.5	128	3.20	1.83	0.37	1.83	0.73	0.73
	100%	274	73	18	0	65.0	128	3.20	1.83	0.37	1.83	0.73	0.73
DWAM3	0	324	86	22	1290.0	0	151	3.78	2.16	0.43	2.16	0.86	0.86
	60%	324	86	22	516.0	36.0	151	3.78	2.16	0.43	2.16	0.86	0.86
	70%	324	86	22	387.0	42.0	151	3.78	2.16	0.43	2.16	0.86	0.86
	80%	324	86	22	258.0	48.0	151	3.78	2.16	0.43	2.16	0.86	0.86
	90%	324	86	22	129.0	54.0	151	3.78	2.16	0.43	2.16	0.86	0.86
	100%	324	86	22	0	60.0	151	3.78	2.16	0.43	2.16	0.86	0.86

**Table 6:** Mix proportion for property regulation of DWAM (kg/m^3^).

Series	Aerogel Volume	Cement	Slag	Silica Fume	DWRA	Aerogel	Water	KH-550	PCE	AEA	RLP	HPMC	PP
DWAM3-A1	80%	324	86	22	258.0	48.0	151	3.78	2.16	0.86	2.16	0.86	0.86
DWAM3-A2	80%	324	86	22	258.0	48.0	151	3.78	2.16	1.30	2.16	0.86	0.86
DWAM3-A3	80%	324	86	22	258.0	48.0	151	3.78	2.16	1.73	2.16	0.86	0.86
DWAM3-A4	80%	324	86	22	258.0	48.0	151	3.78	2.16	2.16	2.16	0.86	0.86

## Data Availability

The original contributions presented in this study are included in the article. Further inquiries can be directed to the corresponding author.
